# Safety, pharmacokinetics and pharmacodynamics of remogliflozin etabonate, a novel SGLT2 inhibitor, and metformin when co-administered in subjects with type 2 diabetes mellitus

**DOI:** 10.1186/2050-6511-14-25

**Published:** 2013-04-30

**Authors:** Elizabeth K Hussey, Anita Kapur, Robin O’Connor-Semmes, Wenli Tao, Bryan Rafferty, Joseph W Polli, Charles D James, Robert L Dobbins

**Affiliations:** 1 GlaxoSmithKline, 5 Moore Drive, Research Triangle Park, NC 27709, USA; 2 Tandem Labs, Durham, NC, USA

**Keywords:** Remogliflozin etabonate, SGLT2 inhibitor, Metformin, Pharmacokinetics, Type 2 diabetes mellitus

## Abstract

**Background:**

The sodium-dependent glucose co-transporter-2 (SGLT2) is expressed in absorptive epithelia of the renal tubules. Remogliflozin etabonate (RE) is the prodrug of remogliflozin, the active entity that inhibits SGLT2. An inhibitor of this pathway would enhance urinary glucose excretion (UGE), and potentially improve plasma glucose concentrations in diabetic patients. RE is intended for use for the treatment of type 2 diabetes mellitus (T2DM) as monotherapy and in combination with existing therapies. Metformin, a dimethylbiguanide, is an effective oral antihyperglycemic agent widely used for the treatment of T2DM.

**Methods:**

This was a randomized, open-label, repeat-dose, two-sequence, cross-over study in 13 subjects with T2DM. Subjects were randomized to one of two treatment sequences in which they received either metformin alone, RE alone, or both over three, 3-day treatment periods separated by two non-treatment intervals of variable duration. On the evening before each treatment period, subjects were admitted and confined to the clinical site for the duration of the 3-day treatment period. Pharmacokinetic, pharmacodynamic (urine glucose and fasting plasma glucose), and safety (adverse events, vital signs, ECG, clinical laboratory parameters including lactic acid) assessments were performed at check-in and throughout the treatment periods. Pharmacokinetic sampling occurred on Day 3 of each treatment period.

**Results:**

This study demonstrated the lack of effect of RE on steady state metformin pharmacokinetics. Metformin did not affect the AUC of RE, remogliflozin, or its active metabolite, GSK279782, although C_max_ values were slightly lower for remogliflozin and its metabolite after co-administration with metformin compared with administration of RE alone. Metformin did not alter the pharmacodynamic effects (UGE) of RE. Concomitant administration of metformin and RE was well tolerated with minimal hypoglycemia, no serious adverse events, and no increase in lactic acid.

**Conclusions:**

Coadministration of metformin and RE was well tolerated in this study. The results support continued development of RE as a treatment for T2DM.

**Trial registration:**

ClinicalTrials.gov, NCT00376038

## Background

Type 2 diabetes mellitus (T2DM) is a chronic disease characterized by deteriorating glycemic control and an associated risk of complications. Evidence from controlled clinical trials suggests that improving glycemic control can substantially reduce the long-term microvascular complications of diabetes [1-5]. Current guidelines recommend that T2DM patients should be initially managed with diet and exercise followed by pharmacological treatment with metformin as the preferred step 1 agent, unless there are contraindications to metformin use. When glycemic goals are not achieved, the dose of metformin is increased or a second agent is added [6,7]. In this treatment algorithm, suitability for combination with metformin becomes a critical concern in developing new antidiabetic agents.

Metformin is a dimethylbiguanide that reduces elevated blood glucose levels primarily through its effects on reducing hepatic glucose production and improving peripheral tissue sensitivity to insulin. Metformin is typically administered with meals and has an oral bioavailability of approximately 40 to 60% [8]. It undergoes extensive renal excretion 3 times the glomerular filtration rate [9] and has a mean plasma elimination half-life between 4.0 and 8.7 hours. There are no clinically relevant metabolic interactions reported with metformin, and it is neither metabolized nor inhibits the metabolism of other drugs [10]. However, there are several transporter related drug interactions, in particular with cationic drugs that have been reported [9]; these typically don’t require a dose adjustment. The main adverse event of clinical concern with metformin is lactic acidosis, a potentially life-threatening side effect that may be associated with high plasma concentrations of metformin and renal insufficiency [11-13].

The low-affinity, high-capacity sodium-dependent glucose co-transporter-2 (SGLT2), which is expressed specifically in the renal proximal tubule [14,15], plays a major role in the reabsorption of glucose by the kidney. SGLT2 has recently gained recognition as a potential therapeutic target for reducing hyperglycemia in T2DM, and several selective SGLT2 inhibitors are being evaluated in the clinic [16-22]. In diabetic animal models, pharmacological inhibition of SGLT2 leads to glucosuria followed by normalization of plasma glucose levels and consequent improvement in insulin resistance [23-25]. This mechanism may provide improvements in both fasting and postprandial hyperglycemia without causing weight gain or other dose-limiting side effects observed with other oral antidiabetic approaches [26].

Remogliflozin etabonate is the prodrug of the highly selective and potent SGLT2 inhibitor, remogliflozin. Administration of remogliflozin etabonate has been shown to increase urinary glucose excretion in a dose-dependent manner in mice and rats and to exhibit antidiabetic efficacy in several diabetic rodent models [27]. Remogliflozin is further metabolized to GSK279782, which is an equally potent inhibitor of SGLT2 [28] but circulates at approximately 20% of the plasma concentrations of remogliflozin; thus GSK279682 is expected to contribute to some of the observed SGLT-2 inhibitor pharmacology. Single oral doses of remogliflozin etabonate up to 1000 mg in healthy subjects and repeated dosing in subjects with T2DM (up to 1000 mg BID for 2 weeks) have been safe and well tolerated [29,30]. Remogliflozin etabonate is intended for use in the treatment of T2DM as monotherapy. Given its mechanism of action, it would be a candidate for combination with metformin and other antidiabetic therapies as well. The osmotic diuresis associated with increased urine glucose excretion provides a potential mechanism for pharmacokinetic drug–drug interactions due to the extensive renal clearance of metformin, although treatment with the diuretic hydrochlorothiazide for 2 weeks had no significant effect on the clearance of metformin in subjects with T2DM [31].

This study was designed to evaluate the effect of remogliflozin etabonate on metformin exposure in T2DM subjects. Secondarily, the effect of metformin on steady state plasma concentrations of remogliflozin etabonate, remogliflozin (active entity) and the active metabolite, GSK279782 was evaluated. Three days of dosing (total of 5 doses) was considered adequate to achieve steady-state conditions for both metformin and remogliflozin. Safety problems that might be related to a pharmacokinetic drug–drug interaction were also monitored.

## Methods

This single-center, Phase 1 study was conducted at Medica Sur Hospital and Clinical Foundation Pharma Unit (CIF-BIOTEC), Mexico. This study was approved by the investigational center ethics committee (Hospital Medical Sur Ethics Committee) and was conducted in accordance with Good Clinical Practice and the principles of the Declaration of Helsinki. All subjects provided their written informed consent before study participation. The study was registered at http://clinicaltrials.gov with the identifier NCT00376038.

### Subjects

Male and female subjects (post-menopausal women or pre-menopausal women with documented hysterectomy or tubal ligation) with documented T2DM (≥3 months), ranging in age from 30 to 64 years and with a body mass index of 22 to 35 kg/m^2^, were eligible for the study. Enough subjects were to be enrolled to ensure completion of at least 12 evaluable subjects. Pre-study screening included a medical history, physical examination, medical and laboratory evaluations, including 12-lead ECG, and a urinary drug screen. Subjects were required to be free of clinically significant medical and laboratory abnormalities, to have glycosylated hemoglobin (HbA1c) <10%, and fasting plasma glucose (FPG) <280 mg/dL, and to be controlled by diet alone or metformin. Standard exclusion criteria concerning blood donation, alcohol and drug use, caffeine intake, and participation in other recent investigational drug studies were applied. In addition, subjects were excluded from participation in the study if they required insulin, had received insulin within the past 3 months, or if they had significant renal disease (as manifested by one or more of the following: creatinine clearance <60 mL/min/1.73 m^2^, urine albumin concentration ≥300 μg/mg of creatinine, or a spot urine sample with a urine protein/creatinine ratio >2.5 mg/mg (a ratio that approximates the common cut off of 3 g of protein in urine per 24 hours to exclude subjects with nephrotic range proteinuria [32]).

### Study design

The study was a randomized, open-label, repeat-dose, two-sequence, cross-over study in subjects with T2DM who were taking metformin or who were drug naive. Before randomization, eligible subjects were stratified on the basis of their pre-entry treatment regimen: metformin or drug naive. Subjects were randomized to receive one of two treatment sequences depicted in Table 1. Each treatment sequence included three treatment regimens [A = metformin 500 mg every 12 hours (MET BID), B = remogliflozin etabonate 500 mg every 12 hours (RE BID), and C = metformin 500 mg + remogliflozin etabonate 500 mg every 12 hours (MET + RE)] administered over three 3-day dosing periods that were separated by two non-treatment intervals of variable duration (minimum of 2 days up to a maximum of 15 days). The last dose of drug for each study period was before breakfast on day 3. Metformin was administered as Glucophage^®^ (Bristol-Myers Squibb, New York, NY) and subjects were allowed to continue taking metformin during the non-treatment interval between the first and second treatment periods. On the evening before each treatment period, subjects were admitted and confined to the clinical site for the duration of the 3-day treatment period. Pharmacokinetic (PK), pharmacodynamic (PD; urine glucose and FPG), and safety (adverse events, vital signs, ECG, clinical laboratory parameters including lactic acid) assessments were performed at check-in and throughout the treatment periods. For each treatment period, the PK sampling occurred on Day 3.

**Table 1:** Treatment sequence regimens

**Treatment sequence**	**Period 1**	**Interval between dosing**	**Period 2**	**Interval between dosing**	**Period 3**
	***3 Days***	***2 to 15 days***	***3 days***	***2 to 15 days***	***3 days***
1	A	Continue metformin only	C	Stop all trial medications	B
2	C	Continue metformin only	A	Stop all trial medications	B

Treatment A (MET BID): Metformin IR 500 mg every 12 hours. Treatment B (RE BID): Remogliflozin etabonate 500 mg every 12 hours. Treatment C (MET + RE BID): Metformin IR 500 mg every 12 hours + remogliflozin etabonate 500 mg every 12 hours. Metformin was administered starting from the morning of Day 1 of Period 1 and stopped after the morning dose on Day 3 of Period 2. For any Treatment Period when remogliflozin etabonate was administered, remogliflozin etabonate dosing was stopped after the morning dose was given on Day 3.

Subjects were asked to refrain from drinking grapefruit juice or eating grapefruit for at least 3 days before the first dose until collection of the final PK sample for each treatment period. Subjects were to abstain from alcohol or caffeine- or xanthine-containing products from up to 24 hours prior to admission until collection of the final blood/urine sample. Subjects who smoked had to be able to abstain from use of tobacco products for the 12-hour PK sampling interval. On days 1–3 of each treatment regimen, while in-house, subjects were fed breakfast, lunch, and dinner as standard meals with identical meals provided on the PK sampling days. Subjects were given 1700 kcal per day, with calories distributed as 55% carbohydrate, 25% fat, and 20% protein. Breakfast was served at approximately 7am and dinner at approximately 7 pm. Subjects were instructed to complete these meals within 30 minutes. Within 15 minutes of completing the meal, the study medications were administered with 240 mL of water per the randomization schedule. Use of the following concomitant medications was allowed if the dosing regimen had been stable for at least 3 months prior to study enrollment: 3-hydroxy-3-methyl-glutaryl-CoA reductase inhibitors, ACE inhibitors, angiotensin receptor blockers, hydrochlorothiazide (dose of ≤25 mg/day), calcium channel blockers, alpha or beta blockers, thyroid hormone (only if TSH in normal range), hormone replacement therapy, inhaled and intranasal corticosteroids, antidepressants (SSRIs only) and multivitamins. Low-dose acetaminophen or ibuprofen (≤1.2 g/day), and any medications prescribed for treatment of adverse events occurring during the study were also allowed. Concomitant medications were not permitted within 4 hours of study drug administration.

### Clinical and laboratory monitoring for safety

For each treatment period, subjects were admitted to the clinical facility on the evening of Day -1 to undergo check-in procedures including a physical examination, 12-lead ECG, vital signs, clinical laboratory tests (chemistry, hematology and urinalysis), lactic acid measurement, fasting blood glucose measurement, alcohol screen, drugs of abuse screen and pregnancy test (if applicable). On each study day morning, a fasting blood sugar measurement was determined by glucose monitor. On Days 1 and 2, vital signs and a 12-lead ECG were recorded. Samples for clinical laboratory measure were also taken on Days 1 and 3. Subjects returned to the clinic 7–10 days following the last dosing day for a follow-up physical examination and laboratory evaluation. During the between-treatment intervals, subjects were provided with glucose monitors to measure fasting blood glucose concentrations; subjects were instructed as to how to recognize and treat symptoms of hypoglycemia. Adverse events were monitored throughout the entire study (randomization to follow-up visit). Any adverse events reported during the study were assessed by the investigator for intensity (mild, moderate, severe) and relationship to the study drug (causality). Where possible, all adverse events were followed until stabilization, resolution, or until the event was otherwise explained.

### Pharmacokinetic assessment

#### Blood sampling

Serial blood (two 2 mL samples for metformin and for remogliflozin etabonate and metabolites) were collected pre-dose, 0.25, 0.5, 0.75, 1, 1.5, 2, 3, 4, 6, 8, and 12 hours post-dose for determination of plasma metformin, remogliflozin etabonate (prodrug), remogliflozin (active entity) and GSK279782 (metabolite) concentrations. All sample times are relative to the time of the administration of the first dose of study medication on Day 3 of each period. Blood samples for metformin were collected into tubes containing EDTA and immediately placed on ice and centrifuged at approximately 3000 rpm for 10 minutes at approximately 4°C. The harvested plasma was separated, frozen and stored at -20°C or lower until analysis for metformin concentrations. Blood samples for remogliflozin etabonate, remogliflozin and GSK279782 were collected into tubes containing potassium-oxalate/ sodium fluoride, placed on ice and centrifuged at approximately 3000 rpm for 10 minutes at approximately 4°C. The harvested plasma was frozen at -70°C until analysis for remogliflozin etabonate, remogliflozin and GSK279782 concentrations.

#### Drug assays

The concentrations of remogliflozin etabonate, remogliflozin, and GSK279782 in deproteinized plasma samples and standards were determined by high-performance liquid chromatography (HPLC) with tandem mass spectrometry (MS/MS) using isotopically labelled internal standards ([^2^H_7_]- remogliflozin etabonate, [^2^H_7_]- remogliflozin and [^2^H_7_]-GSK279782 as previously described [28].

The concentrations of metformin in plasma were determined by HPLC-MS/MS using a [^2^H_6_]-metformin isotopically labelled internal standard. Plasma proteins from a 50 mL plasma aliquot were precipitated using acetonitrile containing the internal standard (200 ng mL-1). Samples were vortex mixed then centrifuged. The resulting supernatant was transferred and mixed with 200 mL of HFBA buffer (water containing 10 mM ammonium acetate and 0.26% (v/v) of heptofluorobutyric acid) prior to injection. HPLC was performed on a Shimadzu LC-10A HPLC system. Chromatography was performed on a MAC-MOD Ace 3 C18, 4.6 × 50 mm column at a flow rate of 1.0 mL min-1. An isocratic mobile phase elution with 82:18 (v/v) HFBA buffer : Acetonitrile was used. Samples were analysed in positive ion mode by Turbo Ionspray LC/MS/MS with a PE/Sciex API 3000. The calibration range was 20 to 5000 ng mL-1. Performance of the method was assessed during a 3 day validation study using quality control samples at 5 concentrations 20, 80, 500, 4000 and 5000 ng mL-1. The average within-run precision [coefficient of variation (CV %)] was <9.6% and the between-run precision CV% was < 4.7%. Similar assay performance was observed during study sample analyses.

#### Pharmacokinetic calculations

PK analyses of plasma concentration–time data of each analyte (i.e., metformin, remogliflozin etabonate, remogliflozin, and GSK279782) were conducted using the noncompartmental Model 200 (for extravascular administration) of WinNonlin Professional Edition version 4.1 (Pharsight Corporation, Mountain View, CA, USA). Actual elapsed time from dosing was used to estimate all individual plasma PK parameters. Values for the following PK parameters were estimated for each analyte, as appropriate, following administration of 3 days dosing of metformin, remogliflozin etabonate, or both.

• C_max_ and t_max_ were the actual observed values.

• AUC_(0–12)_ or AUC_(0–last)_ was calculated by a combination of linear and logarithmic trapezoidal methods. The linear trapezoidal method was used for all incremental trapezoids arising from increasing concentrations and the logarithmic trapezoidal method was used for those arising from decreasing concentrations.

### Pharmacodynamic assessment

#### Plasma PD

FPG concentrations were collected on Day -1, 1, 2 and 3. Changes in plasma glucose from baseline (Day 1) to Day 2 and 3 were calculated.

#### Urine PD

Urine was collected on Days 1 to 3 of each treatment period, and urine glucose concentrations were analyzed for the following intervals: 0–4 hours, 4–8 hours, 8–12 hours and 12–24 hours. The quantity of glucose and creatinine excreted in urine was determined by multiplying the urine glucose or creatinine concentration for each time interval by the volume of urine for the corresponding collection interval. The total 24-hour quantity of glucose excreted in urine on Day 2 was calculated by adding the amounts collected during each interval. Urine glucose and creatinine amounts were summarized for each collected interval and for the total 24-hour collection period.

Creatinine clearance (CLcr) was calculated on Day 2 and used to determine the percent of filtered glucose load excreted in urine. By using the urine collections on Day 2, CLcr was calculated as follows:

$$\begin{array}{rl} \text{Clcr}= & \text{total}\;\text{amount}\;\mathrm{of}\;\text{urine}\;\text{creatinine}\;0-\text{xxh}\;\text{interval}\\ & /\text{nearest}\;\text{associated}\;\text{serum}\;\text{creatinine}\\ & =\left(\left(\text{urine}\;\text{creatinine}\left(\mathrm{mg}/\mathrm{xx}\;\text{hours}\right)\right)\right)\\ & \left(\left(/\text{serum}\;\text{creatinine}\left(\mathrm{mg}/\mathrm{dL}\right)\right)*100\;\mathrm{mL}/\mathrm{dL}\right)\\ & /\left(\mathrm{xx}*60\right)\left(\text{minutes}/\mathrm{xx}\;\text{hours}\right), \end{array}$$

where urine creatinine (mg/xx hours) is the amount of urine excreted in a xx-hour period. Urine creatinine was calculated by multiplying the urine creatinine concentration by the urine volume (mL) for a 0-xxh time interval as follows:

$$\begin{array}{l} \text{urine}\;\text{creatinine}\;\left(\mathrm{mg}/\mathrm{xx}\;\text{hours}\right)\\ \;=\left(\text{urine}\;\text{creatinine}\;\text{concentration}\left(\mathrm{mg}/\mathrm{dL}\right)*\right)\\ \;\left(\text{interval}\;\text{volume}\left(\mathrm{mL}\right)\right)/100\left(\mathrm{mL}/\mathrm{dL}\right). \end{array}$$

CLcr was reported in mL/minutes on Day 2 for collection intervals 0–4 hours, 4–8 hours, 8-12 hours, 12–24 hours, and the total daily interval of 0–24 hours.

The serum creatinine concentration used for the above calculations was the pre-dose value for the same day as the urine collection or the one closest to the day of urine collection if no serum creatinine was collected on that day.

#### Percent of filtered glucose excreted in the urine

Percent of filtered glucose excreted in the urine was estimated for all collection intervals on Day 2 as follows:

$$\begin{array}{l} \left(\text{Glucose}\;\text{Amount}\;\text{Excreted}\right)/\left(\mathrm{Clcr}*\mathrm{PG}*\text{Time}\;\text{Interval}\right)\;\mathrm{or}\;\\ \left[\text{Urine}\;\text{glucose}\;\left(\mathrm{mg}/\mathrm{dL}\right)*\text{Serum}\;\text{Creatinine}\;\left(\mathrm{mg}/\mathrm{dL}\right)\right]/\\ \left[\text{urine}\;\text{creatinine}\;\left(\mathrm{mg}/\mathrm{dL}\right)*\mathrm{PG}\left(\mathrm{mg}/\mathrm{dL}\right)\right] \end{array}$$

where glucose amount excreted is the amount of glucose excreted during the xx-hour period, and CLcr is calculated for the xx-hour time interval. PG is the plasma glucose concentration reported closest to the midpoint of the time interval. Because only pre-dose PG was collected in this study, the pre-dose PG on Day 2 was used. Time interval is the number of minutes of urine collection for that interval.

Total fluid intake, urine volume, and fluid balance (intake minus output) were summarized over the 0–24-hour interval of Day 1 and Day 2 and the 0–12-hour interval of Day 3 of each treatment period.

### Statistical analysis

The sample size was based on the primary endpoint, metformin AUCs_(0–12)_ and assumed a within-subject standard deviation of 0.15 [33,34] for natural log-transformed AUC . Using the two one-sided *t*-test [35] at type I error α=0.05 under a crossover design, 12 subjects should provide at least 90% power to demonstrate lack of an interaction if the ratio of test to reference is truly 1 and the equivalence criteria for the 90% confidence interval (CI) is 0.8–1.25.

Safety and PD parameters were summarized using descriptive statistics. Analyses of steady-state plasma metformin AUC_(0–12)_ and C_max_ were conducted with metformin alone as the reference treatment. A mixed effect model with ln(AUC(0–12)) as the dependent variable; treatment, period and sequence as fixed effects; and subject-within-sequence as a random effect was used to estimate the treatment difference and its associated 90% CI on the log scale. The PROC MIXED from SAS (Version 8.2, Cary, NC, USA) was used to fit the model. The estimates and the 90% CI were exponentiated in order to obtain the ratio of geometric means and its CI. The assumptions underlying the model were assessed by visual inspection of residual plots.

Similar analyses were performed for the secondary PK endpoints for remogliflozin etabonate, remogliflozin and its metabolite, with and without metformin. T_max_ was analysed non-parametrically using Hodges–Lehmann method [36,37].

## Results

Thirteen subjects (7 females [54%] and 6 males [46%]) were randomized and completed the study. Of these 13 subjects, 10 subjects were being treated with metformin before study entry and three subjects were drug naive before study entry. The median age was 54 years (range 38 to 62 years); the median BMI was 29 kg/m^2^ (range 22.5 to 34.3 kg/m^2^); mean fasting plasma glucose at baseline was 7.21 mmol/L (SD 1.77; range 4.8 to 10.9 mmol/L). All subjects were Hispanic or Latino.

### Pharmacokinetics

**Figure 1:**
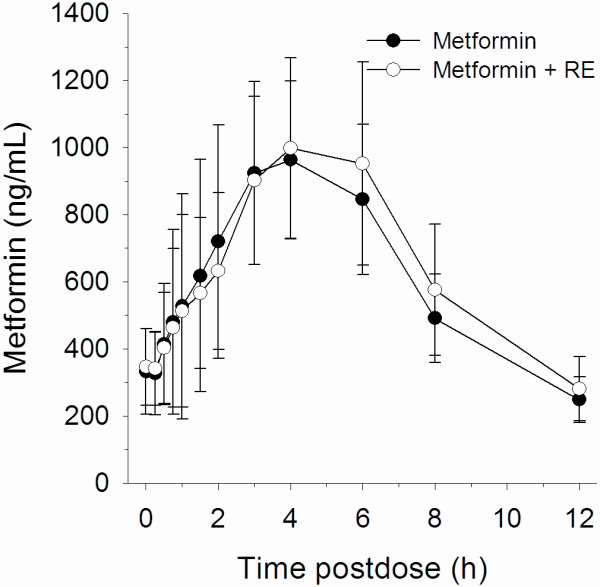
Mean metformin concentration (and standard deviation) vs. time profiles with and without remogliflozin etabonate, n = 13.

The summary data of PK parameters for metformin, remogliflozin etabonate, remogliflozin and GSK279782 are presented in Table 2. The primary PK objective was to demonstrate a lack of effect of remogliflozin etabonate on the PK parameters of metformin. Results from the primary comparison, are summarized in Table 3 and mean concentration vs time profiles are shown in Figure 1. There was no effect of remogliflozin etabonate on metformin PK parameters.

**Table 2:** Summary of plasma metformin, remogliflozin etabonate, remogliflozin, and GSK279782 PK parameters

**Metformin PK parameter**	**MET BID**	**MET + RE BID**
	**N = 13**	**N = 13**
AUC_(0–12)_ (h.ng/mL)	7141.3 (24)	7520.8 (27)
C_max_ (ng/mL)	1018.2 (26)	1025.3 (25)
t_max_ (h)	4.0 (1.0 - 6.0)	4.0 (1.0–6.0)
**Remogliflozin etabonate (prodrug) PK Parameter**	**RE BID**	**MET + RE BID**
	**N = 12-13**^a^	**N = 12-13**^a^
AUC_(0–last)_ (h.ng/mL)	98.9 (69)	102.1 (49)
C_max_ (ng/mL)	79.5 (107)	67.7 (77)
t_max_ (h)	3.0 (1.0–4.0)	3.0 (1.0–6.0)
**Remogliflozin (active entity) PK Parameter**	**RE BID**	**MET + RE BID**
	**N = 13**	**N = 13**
AUC_(0–12)_ (h.ng/mL)	6814.3 (33)	6425.9 (33)
C_max_ (ng/mL)	2688.6 (52)	2124.6 (63)
t_max_ (h)	3.0 (1.0–4.0)	3.0 (1.0 - 6.0)
**GSK279782 (active metabolite) PK Parameter**	**RE BID**	**MET + RE BID**
	**N = 13**	**N = 13**
AUC_(0–12)_ (h.ng/mL)	1527.9 (37)	1472.9 (36)
C_max_ (ng/mL)	462.8 (39)	361.9 (38)
t_max_ (h)	4.0 (1.0–4.0)	4.0 (1.0–8.0)

Values are geometric mean (%CVb) for each parameter, except for t_max_ which is median (range). PK, pharmacokinetic; MET BID, metformin 500 mg every 12 hours; MET + RE BID, metformin 500 mg + remogliflozin etabonate 500 mg every 12 hours; RE BID, remogliflozin etabonate 500 mg every 12 hours.

^a^ AUC not evaluable for one subject.

**Table 3:** Statistical comparisons of PK parameters of metformin, remogliflozin etabonate, remogliflozin, and GSK279782 with and without remogliflozin etabonate

**Compound**	**PK parameter**	**Treatment comparison**	**Point estimate (GLSM Ratio)**	**90% CI**
Metformin	AUC(0–12) ^[1]^	MET + RE / MET	1.05	(0.98, 1.12)
	Cmax	MET + RE / MET	1.01	(0.92, 1.10)
Remogliflozin etabonate (prodrug)	AUC_(0-last)_	MET + RE / RE	1.00	(0.77, 1.29)
	C_max_	MET + RE / RE	0.85	(0.54, 1.35)
Remogliflozin (active entity)	AUC_(0–12)_	MET + RE / RE	0.94	(0.86, 1.04)
	C_max_	MET + RE / RE	0.79	(0.60, 1.05)
GSK279782 (active metabolite)	AUC_(0–12)_	MET + RE / RE	0.96	(0.92, 1.01)
	C_max_	MET + RE / RE	0.78	(0.67, 0.91)

^[1]^ primary comparison; MET + RE, metformin 500 mg + remogliflozin etabonate 500 mg every 12 hours; GLSM : Geometric least-squares mean.

**Figure 2:**
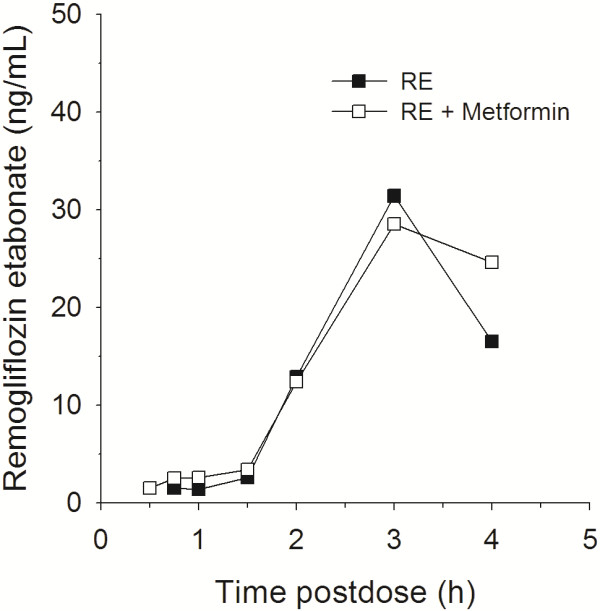
**Median remogliflozin etabonate (prodrug) concentration vs. time profiles with and without metformin, n = 13. **(Median data is presented in this plot because the majority of the samples were below the lower limit of quantification).

**Figure 3:**
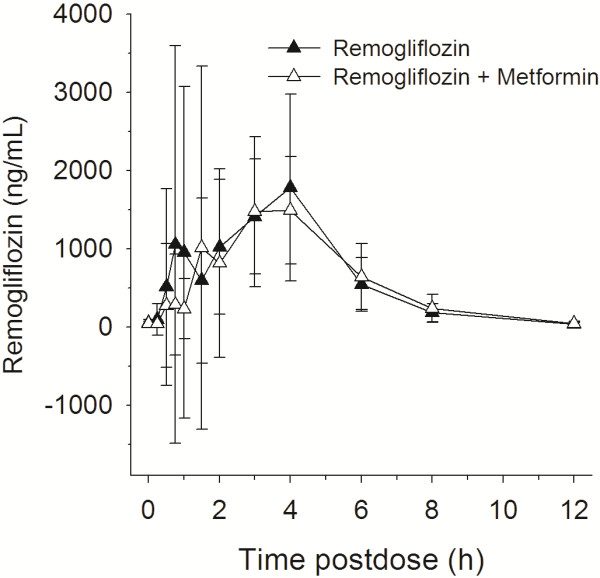
Mean remogliflozin (active entity) concentration (and standard deviation) vs. time profiles with and without metformin, n = 13.

**Figure 4:**
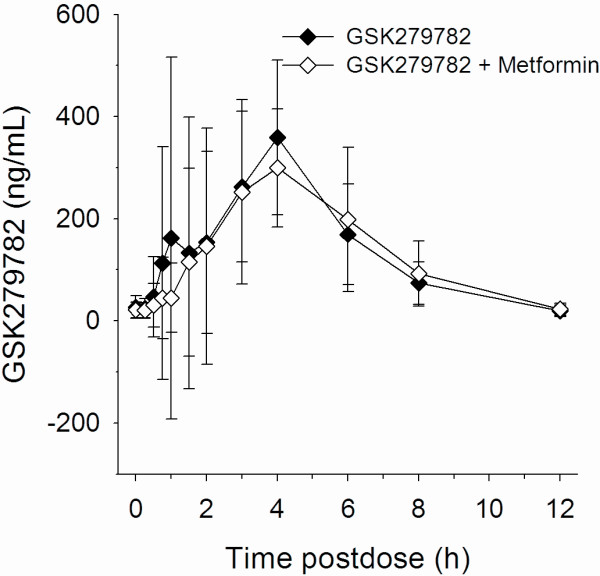
Mean GSK279782 (active metabolite) concentration (and standard deviation) vs. time profiles with and without metformin, n = 13.

One of the secondary objectives included a comparison of PK parameters for remogliflozin etabonate, remogliflozin and GSK279782 after treatment with remogliflozin etabonate alone and with MET + RE. A summary of these results is presented in Table 3 and concentration vs. time profiles are provided in Figures 2, 3, 4. There were no effects of metformin on the AUC of remogliflozin etabonate, remogliflozin, or its metabolite, GSK279782. However, C_max_ was lower with the combination. For C_max_, on average, there was a decrease of 21% in remogliflozin and a decrease of 22% in GSK279782 with MET + RE compared to remogliflozin etabonate alone. The 90% CI indicates that the true difference lies between a decrease of 40% and an increase of 5% for remogliflozin and between a decrease of 33% and 9% for GSK279782.

### Pharmacodynamics

#### Fasting plasma glucose

**Figure 5:**
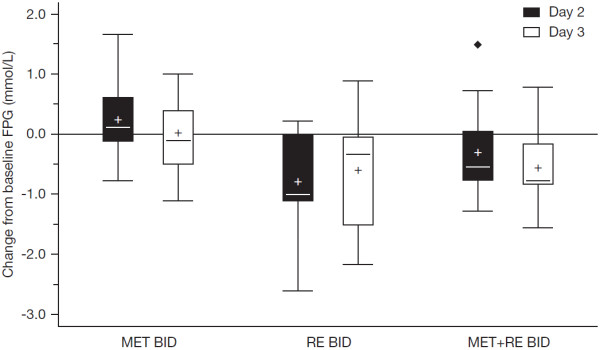
**Fasting plasma glucose concentration (FPG; mmol/L) – Change from baseline (pre- dose on Day 1 of each treatment period). **MET BID, metformin 500 mg every 12 hours; RE BID, remogliflozin etabonate 500 mg every 12 hours; MET + RE BID, metformin 500 mg + remogliflozin etabonate 500 mg every 12 hours. Mean (and standard deviation) baseline FPG values for each treatment period: MET BID: 6.72 (1.88); RE BID, 6.98 (2.06); MET + RE BID, 6.42 (1.15).

A summary of the FPG concentration data by treatment period and study day is presented in Figure 5. When the changes in fasting plasma glucose concentrations from baseline (pre-dose on Day 1) to Day 2 and Day 3 were considered for the three treatment periods, it appeared that the fasting glucose concentrations remained relatively stable during the MET BID period, whereas small decreases were observed during both the RE BID and MET + RE BID treatment periods.

#### Urinary glucose excretion and percent of filtered glucose excreted

Mean cumulative 24-hour urinary glucose excretion was approximately 500 mmol following treatment with RE BID or MET + RE BID (Day 2), whereas MET BID had relatively no effect on urine glucose output (Table 4). The effect of remogliflozin etabonate on urine glucose excretion was not diminished by co-administration with metformin. The greatest increase in urine glucose excretion was evident within the first 4 hours of dosing following both remogliflozin etabonate regimens. The 24-hour creatinine clearance on Day 2 was comparable across the three treatment periods and was approximately 110 mL/min. During the RE BID and MET + RE BID periods, mean and median values for the percent of filtered glucose excreted in the urine ranged from 43% up to 68% during the individual collection intervals, with a mean of approximately 50% for the combined 24 hour collection for both remogliflozin etabonate containing regimens compared to 1.4% with metformin alone (Table 5).

**Table 4:** Summary of 24-hour urine glucose (mmol) on day 2 by treatment

	**Met BID**	**RE BID**	**Met + RE BID**
	**N = 13**	**N = 13**	**N = 13**
Mean (SD)	13.6 (13.4)	528 (130)	458 (98)
Median	10.9	497	485
Min, Max	1.1, 43.9	384, 796	242, 573

**Table 5:** Summary of percent filtered glucose excreted in urine on day 2 by treatment

	**Met BID**	**RE BID**	**Met + RE BID**
	**N = 13**	**N = 13**	**N = 13**
Mean (SD)	1.41 (1.52)	51.3 (7.02)	48.7 (9.87)
Median	0.95	51.8	49.3
Min, Max	0.10, 4.71	38.4, 61.3	35.7, 67.9

#### Fluid balance

Total fluid intake, total urine volume, and fluid balance data for the 24-hour collection intervals on Days 1 and 2 and the initial 12-hour collection interval on Day 3 were compared by treatment. On Days 1 and 2, mean total 24-hour fluid intake ranged from approximately 2500 mL to 3000 mL across the three treatment periods. During the 12-hour collection period on Day 3, mean fluid intake ranged from approximately 1800 to 2200 mL for any one treatment period. Because fluid intake was less than total urine volume throughout all treatment periods, mean fluid balance values were considered negative during most intervals. On Day 1, fluid balance (median, range) appeared more negative on RE BID (-1145 mL, -1630 to +335 mL) and MET + RE BID (-1200 mL, -2395 to -90 mL) compared to MET BID (-775 mL, -2280 to +400 mL). Fluid balance neutrality seemed to be reached on Day 3 for all drug regimens.

### Safety and tolerability

There were no serious adverse events reported. The only adverse event considered related to study drug was hypoglycemic symptoms reported by 2 subjects, one event with metformin alone and one with MET + RE. However, plasma glucose measurements were unfortunately not performed to confirm hypoglycemia. In both cases, the symptoms of hypoglycemia were considered mild in intensity. The events were reported in the time before scheduled meals; the symptoms resolved with provision of food, and did not require a change in study drug. Back pain and headache were the only events reported by more than one subject during any treatment period (reported during MET BID by 2 different subjects). All adverse events are summarized in Table 6.

**Table 6:** Summary of adverse events by treatment

**Preferred term**	**MET BID**	**RE BID**	**MET + RE BID**
	**N = 13**	**N = 13**	**N = 13**
	**n (%)**	**n (%)**	**n (%)**
**Any Event**	**5 (38%)**	**2 (15%)**	**7 (54%)**
Headache	2 (15%)	0	1 (8%)
Back pain	2 (15%)	0	0
Muscle spasms	1 (8%)	1 (8%)	0
Hypoglycemia	1 (8%)	0	1 (8%)
Neck pain	1 (8%)	0	0
Osteoarthritis	0	0	1 (8%)
Abdominal pain upper	0	1 (8%)	0
Dyspepsia	0	0	1 (8%)
Toothache	1 (8%)	0	0
Dizziness	1 (8%)	0	0
Fatigue	1 (8%)	0	0
Nasopharyngitis	0	0	1 (8%)
Wound	0	0	1 (8%)
Rash	0	0	1 (8%)

**Figure 6:**
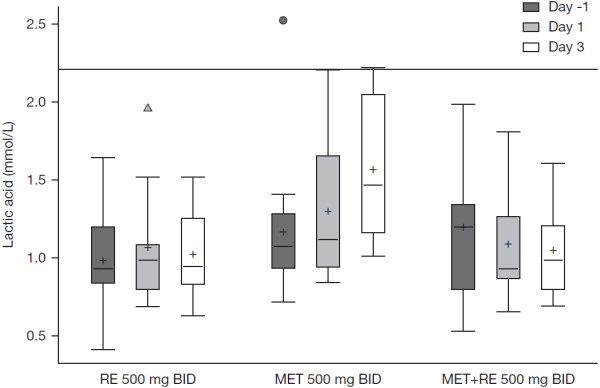
**Lactic acid concentration by treatment (normal range of 0.5 to 2.2 mmol/L). **MET BID, metformin 500 mg every 12 hours; RE BID, remogliflozin etabonate 500 mg every 12 hours; MET + RE BID, metformin 500 mg + remogliflozin etabonate 500 mg every 12 hours.

No clinically significant changes in laboratory parameters or vital signs were reported for any treatment regimen. As an increased exposure to metformin can result in lactic acidosis, lactic acid levels were measured. While there were no instances of lactic acidosis, a trend toward increasing lactic acid was observed with metformin monotherapy relative to regimens including remogliflozin (Figure 6).

## Discussion

Despite the availability of multiple classes and combinations of antihyperglycemic agents, the clinical management of T2DM is currently suboptimal, with the majority of patients failing to achieve and maintain target glycemic levels in practice [38]. Consequently, there is a continued need for novel therapeutic approaches, particularly those with complementary modes of action that will enable further improvement of glycemic control.

Remogliflozin etabonate, by inhibiting glucose reabsorption, offers a potential treatment for T2DM as monotherapy and in combination with existing therapies. Remogliflozin etabonate is being developed for use for the treatment of T2DM as monotherapy, and in combination with existing therapies including metformin. In this study, no effect of remogliflozin etabonate on metformin PK parameters was observed. The findings from this study are consistent with the reported lack of inhibition by remogliflozin etabonate, remogliflozin, and GSK279782 on a panel of metabolic enzymes and transporters, including organic cation transporters involved with metformin renal secretion [39].

This study was not adequately powered to test the effect of metformin on remogliflozin etabonate PK parameters. Metformin did not appear to affect the AUC of remogliflozin etabonate, remogliflozin and its metabolite; however, C_max_ was lower after the co-administration of remogliflozin etabonate and metformin than with remogliflozin etabonate alone. Under the conditions of this study, the peak plasma concentration of remogliflozin considerably exceeded the concentration required for full inhibition of the SGLT2 transporter. However, it is possible that a clinically significant decrease would be observed when administering the combination if low doses of remogliflozin etabonate or considerably higher doses of metformin were given.

As expected on the basis of its pharmacological properties, the administration of remogliflozin etabonate with or without metformin greatly increased urine glucose excretion and the percent of filtered glucose excreted in the urine. The evidence of pharmacological effect was seen within the first 4 hours of dosing with remogliflozin etabonate and sustained while on treatment. Co-administration of metformin with remogliflozin etabonate did not diminish the glucosuric effect of remogliflozin etabonate. Only small changes in fasting glucose concentration were observed during both the RE BID and MET + RE BID treatment periods for this cohort of subjects with good glucose control. Mean fasting glucose concentrations were <7 mmol/L on Day -1 of each treatment period, leaving little room for substantial improvement.

Concomitant administration of remogliflozin etabonate with metformin for 3 days was well tolerated in subjects with T2DM. Hypoglycemia was the only adverse event that was considered related to study drug (and occurred with metformin alone, as well as with the combination). However, neither case was confirmed with plasma glucose concentrations. Antidiabetic treatments that increase urine glucose may increase risk of urinary tract infections (UTIs); however, no documented UTIs were observed over the limited duration of remogliflozin etabonate treatment in this study. Mean lactate concentrations showed an increase or increasing trend during the three day MET BID treatment period. In contrast, mean lactate concentrations are unchanged or decreased slightly during RE BID and MET + RE BID periods. Potential mechanisms to explain the decreased lactate concentrations include reduced glucose concentrations with less production from glycolysis, enhanced extraction of lactic acid by the liver for gluconeogenesis or increased clearance of lactic acid by the kidney. No symptoms suggestive of lactic acidosis occurred during the study.

## Conclusions

In summary, the findings of this study do not indicate a safety concern when multiple oral doses of remogliflozin etabonate 500 mg are administered with metformin 500 mg BID in the intended patient population. Because remogliflozin etabonate does not affect the PK profile of metformin, there is a low risk for adverse events resulting from a PK drug interaction and increased metformin exposure. The approximate 20% decline in remogliflozin Cmax under conditions of coadministration is likely a reflection of the 15% decline in the Cmax of the prodrug (RE) when given with metformin (Table 3). It appears that metformin reduces the Cmax of RE without an effect on RE AUC, suggesting a change in the shape of the 12-hour, steady state, concentration-time profile. Even though the confidence interval is wide for the prodrug Cmax point estimate (0.54, 1.35) and contains 1.0, it is plausible that coadministration of metformin altered GI motility enough to affect the absorption or hydrolysis of RE resulting in a lower Cmax of RE. The lower Cmax values for remogliflozin and GKS279782 following dosing with metformin collectively support this conclusion since they are downstream metabolites of RE.

Although administration with metformin resulted in a 21% reduction in C_max_, the PD properties of remogliflozin etabonate were not altered when administered with metformin. There was an indication that remogliflozin etabonate alone improves plasma blood glucose by increasing the excretion of urine glucose, and this effect by remogliflozin etabonate was not impaired by the co-administration of metformin. Future studies in a larger patient population are warranted to definitively test the safety and efficacy of remogliflozin etabonate in combination with metformin in patients with T2DM who have not achieved the desired glycemic target.

## Competing interests

At the time of study, EKH, AK, ROCS, WT, BR, JWP, CJ, and RLD are employees of GlaxoSmithKline.

## Authors’ contributions

EKH, AK, ROCS, WT, BR, JWP, CJ, and RLD participated in the design of the study, its co-ordination and performed the statistical analysis. All authors were involved in critically revising the drafts of the manuscript, and read and approved the final manuscript.

## Pre-publication history

The pre-publication history for this paper can be accessed here:

http://www.biomedcentral.com/2050-6511/14/25/prepub
